# SUPPORT Tools for evidence-informed health Policymaking (STP) 5: Using research evidence to frame options to address a problem

**DOI:** 10.1186/1478-4505-7-S1-S5

**Published:** 2009-12-16

**Authors:** John N Lavis, Michael G Wilson, Andrew D Oxman, Jeremy Grimshaw, Simon Lewin, Atle Fretheim

**Affiliations:** 1 Centre for Health Economics and Policy Analysis, Department of Clinical Epidemiology and Biostatistics, and Department of Political Science, McMaster University, 1200 Main St. West, HSC-2D3, Hamilton, ON, Canada L8N 3Z5; 2 Health Research Methodology PhD Program and Department of Clinical Epidemiology and Biostatistics, 1200 Main St. West, HSC-2D1 Area, Hamilton, ON, Canada L8N 3Z5; 3 Norwegian Knowledge Centre for the Health Services, P.O. Box 7004, St. Olavs plass, N-0130 Oslo, Norway; 4 Clinical Epidemiology Program, Ottawa Health Research Institute, Administration Building, Room 2-017, 1053 Carling Ave., Ottawa, ON, Canada K1Y 4E9; 5 Norwegian Knowledge Centre for the Health Services, P.O. Box 7004, St. Olavs plass, N-0130 Oslo, Norway; Health Systems Research Unit, Medical Research Council of South Africa; 6 Norwegian Knowledge Centre for the Health Services, P.O. Box 7004, St. Olavs plass, N-0130 Oslo, Norway; Section for International Health, Institute of General Practice and Community Medicine, Faculty of Medicine, University of Oslo, Norway

## Abstract

*This article is part of a series written for people responsible for making decisions about health policies and programmes and for those who support these decision makers*.

Policymakers and those supporting them may find themselves in one or more of the following three situations that will require them to characterise the costs and consequences of options to address a problem. These are: 1. A decision has already been taken and their role is to maximise the benefits of an option, minimise its harms, optimise the impacts achieved for the money spent, and (if there is substantial uncertainty about the likely costs and consequences of the option) to design a monitoring and evaluation plan, 2. A policymaking process is already underway and their role is to assess the options presented to them, or 3. A policymaking process has not yet begun and their role is therefore to identify options, characterise the costs and consequences of these options, and look for windows of opportunity in which to act. In situations like these, research evidence, particularly about benefits, harms, and costs, can help to inform whether an option can be considered viable. In this article, we suggest six questions that can be used to guide those involved in identifying policy and programme options to address a high-priority problem, and to characterise the costs and consequences of these options. These are: 1. Has an appropriate set of options been identified to address a problem? 2. What benefits are important to those who will be affected and which benefits are likely to be achieved with each option? 3. What harms are important to those who will be affected and which harms are likely to arise with each option? 4. What are the local costs of each option and is there local evidence about their cost-effectiveness? 5. What adaptations might be made to any given option and could they alter its benefits, harms and costs? 6. Which stakeholder views and experiences might influence an option's acceptability and its benefits, harms, and costs?

## About STP

*This article is part of a series written for people responsible for making decisions about health policies and programmes and for those who support these decision makers. The series is intended to help such people to ensure that their decisions are well-informed by the best available research evidence. The SUPPORT tools and the ways in which they might can be used are described in more detail in the Introduction to this series *[1]. *A glossary for the entire series is attached to each article *(see Additional File 1). *Links to Spanish, Portuguese, French and Chinese translations of this series can be found on the SUPPORT website http://www.support-collaboration.org. Feedback about how to improve this tool and others in this series is welcome, and should be sent to: *STP@nokc.no.

## Scenarios

*Scenario 1: You are a senior civil servant and will be submitting a brief report to the Minister of Health regarding the evidence to support a number of options to address a high-priority problem. You are concerned about whether the current draft of the report includes a reasonable set of options. You are also concerned about whether the report addresses the likely questions about each option that can reasonably be answered through the use of research evidence*.

*Scenario 2: You work in the Ministry of Health and are preparing a brief report about options to address a high-priority problem that you have been examining in great depth. All that you have been told is that the report should present three options and focus only on what the research evidence says about each option*.

*Scenario 3: You work in an independent unit that supports the Ministry of Health in its use of evidence in policymaking. You are preparing a detailed research report for the Ministry of Health about what is known and not known about options to address a high-priority problem. You have been told what options to examine but want guidance on the types of research evidence about each option that could be used to inform a choice between options*.

## Background

**Figure 1:**
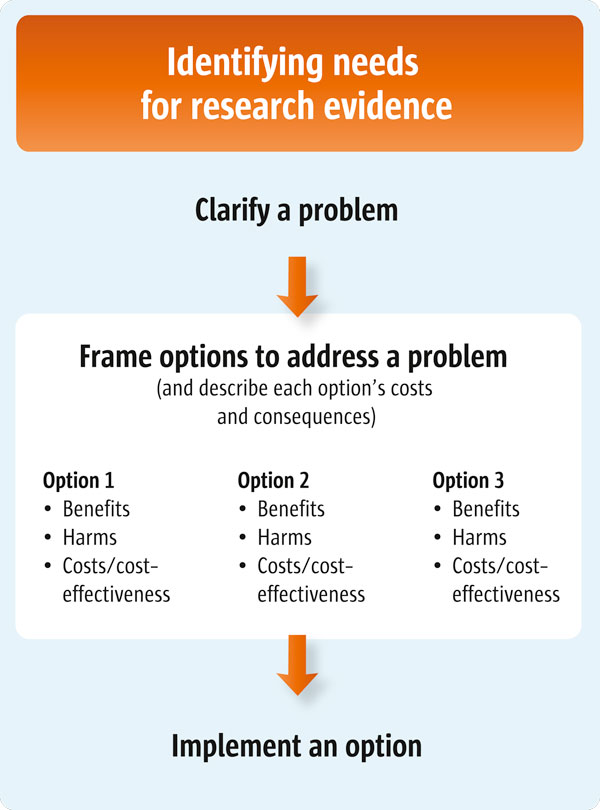
**Clarifying evidence needs**.

For policymakers (Scenario 1), this article suggests a number of questions that they might ask their staff to consider when preparing a brief report about options to address a problem. For those who support policymakers (Scenarios 2 and 3), this article suggests a number of questions to guide the identification of options and the characterisation of each option's costs and consequences. This article is the second of three articles about clarifying evidence needs (see also Articles 4 and 6 [2,3]). Figure 1 outlines the processes involved in clarifying these evidence needs.

Policymakers and those supporting them may find themselves in one or more of the following three situations that will require them to characterise the costs and consequences of options to address a problem. Firstly, a problem may already have been framed in a particular way, an option selected to address the problem, and a political constituency mobilised to support such decisions. (A variant of this situation is that an option may be selected first and then a problem identified later as a motivation for the option.) In this circumstance, the best option for those who support policymakers is to assist them in identifying how to maximise the benefits of the option, how to minimise its harms, and how to optimise the impacts achieved for the money spent. In addition, if there is substantial uncertainty about the likely costs and consequences of an option, a monitoring and evaluation plan can be designed to ensure that policymakers will have the right information to hand at a defined point in the future. This will help them to decide whether a policy should be left unchanged, modified or repealed (or whether a programme should be continued, modified or discontinued). Article 18 in this series describes how to develop a monitoring and evaluation plan [4].

Secondly, policymakers may find themselves in a scenario in which they are actively engaged in a policymaking process. This may mean that policymakers will need to participate in events in which options are being actively debated, meet with 'policy entrepreneurs' who want to persuade them to endorse a particular option, and respond to feedback about the operation of an existing policy or programme [5]. In this second scenario, they will need to assess the options presented to them, the degree of attention being given to the problem that the option is meant to address, and any political events that may present a window of opportunity during which particular actions could be undertaken.

Thirdly, policymakers may find themselves in a situation where they will have more open, strategic opportunities in which they are able to define a problem, to identify options, to characterise the costs of the options and their consequences, and to look for windows of opportunity to undertake preferred actions. Such opportunities are rare and calculations about when to act will need to be strategic.

A policy or programme can be deemed to be an appropriate solution if it is technically feasible, fits within dominant values and the current provincial/national mood, and is acceptable in terms of budget workability and the likely degree of political support or opposition [5]. Research evidence can form part of this mix in several ways and help to determine the following details about a chosen option:

• Whether it is technically feasible - for example, an option's benefits may have been shown to be substantial and its harms acceptably low. Alternatively, the key elements of the policy or programme may have been shown to be consistent with those elements critical to the success of the option in other settings

• Whether it fits with dominant values and the current national mood and is acceptable in terms of likely political support or opposition - interviews with stakeholders, for example, may reveal whether or not it is perceived to be acceptable, and

• Whether it is acceptable in terms of its budget impact (and whether its value for money has been demonstrated)

Interviews with policymakers have confirmed that they place a high value on research evidence about the benefits, harms and costs of options [6].

## Questions to consider

The following questions can guide how to identify options and characterise their costs and consequences:

1. Has an appropriate set of options been identified to address a problem?

2. What benefits are important to those who will be affected and which benefits are likely to be achieved with each option?

3. What harms are important to those who will be affected and which harms are likely to arise with each option?

4. What are the local costs of each option and is there local evidence about their cost-effectiveness?

5. What adaptations might be made to any given option and could they alter its benefits, harms and costs?

6. Which stakeholder views and experiences might influence an option's acceptability and its benefits, harms, and costs?

### 1. Has an appropriate set of options been identified to address a problem?

Initial work should focus broadly on the options that could affect the problem identified. Creative thinking about this topic can be encouraged by identifying options that affect either or both of the following:

• The provision of a cost-effective programme, service or drug, and

• The health system arrangements that determine whether cost-effective programmes, services or drugs are provided to those who need them

Policymakers and other stakeholders with clinical backgrounds often focus largely on issues related to programmes, services or drugs. But at the same time they also tend to neglect concerns related to the health system arrangements that are needed to ensure a high coverage rate for the very same programmes, services or drugs that matter both to them and to consumers. These health system arrangements may include:

• Delivery arrangements: such as who the programme, service or drug is targeted at; who it is provided by; where the care is provided and what information and communication technology is used to provide it; and the safety and quality systems used

• Financial arrangements: such as who finances the relevant parts of the system, programme, service or drug; how organisations are funded to deliver the programme, service or drug; how professionals are remunerated to provide these; how patients/consumers are presented with incentives to use it; and how resources are allocated to it, and

• Governance arrangements: such as who has the policy, organisational, commercial and professional authority and accountability for those parts of the health system that could play a role in addressing the problem

A key next step is then a consideration of whether these elements can stand alone as options or whether they can be bundled together to form new options appropriate to specific local contexts.

Policymakers may be able to identify existing frameworks that enable the identification of policy or programme options. These frameworks may be the focus of reports in their own right. For example, the Chronic Care Model provides a framework for considering how contributions to effective chronic care can be made through self-management support, decision support, delivery system design, clinical information systems, the health system, and the community more generally [7]. Alternatively, frameworks may be embedded in systematic reviews or in overviews of systematic reviews if they are used to structure the search for, and presentation of, research evidence. For example, an overview of systematic reviews provides a framework for addressing challenges related to human resources for health [8]. One dimension of this framework addressed the training, regulatory and financial mechanisms (i.e. the policy and programme options) that could be employed. The second dimension addressed the supply, distribution, efficient use and performance of healthcare providers (i.e. the option's potential consequences). However, multiple competing frameworks may exist, and there is often no empirical evidence to support the use of one framework over another. Moreover, like the options they are meant to help to identify, frameworks may not be mutually exclusive.

Table 1 provides an example of how the teams supporting the widespread use of artemisinin-based combination therapy in Africa identified relevant options and then approached the characterisation of their costs and consequences, using Questions 2 to 6 listed below as prompts.

**Table 1:** Supporting the widespread use of artemisinin-based combination therapy to treat malaria

The Evidence-Informed Policy Networks (EVIPNet) in ten sub-Saharan African countries described the costs and consequences of three options considered viable in these countries for the support of the widespread use of artemisinin-based combination therapy to treat malaria. The impetus for these activities was the 2006 WHO guidelines on malaria treatment which endorsed artemisinin-based combination therapy (ACT) to treat uncomplicated falciparum malaria [13]. In order to support the widespread use of ACT, national governments in regions with seasonal or endemic malaria had to determine whether to confirm or change:	
• *Delivery arrangements*: including who should dispense ACT (when, where and how), and who should be involved in surveillance, pharmacovigilance and the diagnosis and treatment of atypical cases	
• *Financial arrangements for patients *(e.g. drug subsidies) *and for prescribers *(amongst others), and	
• *Financial arrangements for patients *(e.g. drug subsidies) *and for prescribers *(amongst others), and	
• *Governance arrangements*: including which ACT and other anti-malarial drugs should be registered and licensed for sale (i.e. which drugs, the dosage regimes, and the packaging required), how they could be marketed, who could prescribe them (and how), who could sell or dispense them (and how), and what safeguards should be applied to protect against counterfeit or substandard drugs	
	
EVIPNet teams from each participating country considered options consisting of different 'bundles' of heath system arrangements. One country, for example, considered:	
• Using community health workers for the presumptive treatment of uncomplicated malaria with ACT (a delivery arrangement)	
• Introducing ACT subsidies within the private sector to support their use (a financial arrangement) and regulating adherence to the subsidy policy (a governance arrangement), and	
• Providing incentives to prescribers (specifically nurses and doctors) for a time-limited period to encourage transition to the new treatment (a financial arrangement)	
	
The teams, consisting of individuals such as those in involved in the second and third scenarios outlined earlier, then approached the task of describing the costs and consequences for each option using Questions 2-6 as prompts.	
	

**Type of information about each option**	**Examples of the nature of the research evidence sought about each option**

Benefits	• People: everyone except groups other than children under five years of age (who were being treated under a separate programme) and pregnant women (whose cases of malaria were considered 'complicated' and hence beyond the remit of this element of the WHO guideline)
	• Option: see above
	• Comparison: status quo
	• Outcomes: both process indicators (e.g. coverage rates achieved) and outcome indicators (e.g. survival)

Potential harms	• As above except for outcomes where process indicators of interest included the adherence of community health workers to non-malaria related guidelines. This was because of a fear that ACT would be provided at the expense of treating other important conditions

Costs and cost-effectiveness	• Costs collected in their own setting
	• Economic evaluation conducted using a societal viewpoint given that policymakers were acting in their role as stewards for the entire health system, and not just as payers for publicly financed programmes, services and drugs

Key elements of the option (how and why it works)	• Policymakers had already invested heavily in community health workers and wanted to know whether the shared attributes of community health workers and lay health workers were sufficient to allow them to expect similar benefits to those achieved only with lay health workers [14]

Views and experiences of stakeholders	• Policymakers were aware that a large proportion of malaria treatments were dispensed by 'medicine sellers' [15] rather than health professionals or lay health workers. They therefore wanted to know more about the views and experiences of these sellers

### 2. What benefits are important to those who will be affected and which benefits are likely to be achieved with each option?

The second stage in framing options involves characterising their costs and consequences. The first step in this second phase is to determine the likely benefits (or positive effects) of each option. Policymakers need to decide which benefits are likely to be important to those who will be affected by the decisions taken. Some of the studies consulted, for example, may focus on issues related to survival while others may address health-related quality of life issues. Still others may focus on 'intermediate' outcomes such as coverage rates for an effective treatment. Policymakers also need to decide whether they are more interested in particular groups of people (e.g. children, adults or the elderly) and particular comparisons (e.g. comparing the option of doing nothing with the option of providing standard care).

The acronym 'POCO' refers to the four key elements that must be considered in order to enable the identification of research evidence about the benefits of particular options, and to ensure that such evidence is used effectively:

• **P**eople (e.g. elderly patients with multiple chronic conditions)

• **O**ption (e.g. case management)

• **C**omparison (e.g. routine care), and

• **O**utcome (e.g. health-related quality of life)

Searches for evidence should be as precise as possible about identifying those option features that are most important to policymakers and other stakeholders. Policymakers should also assess the extent to which the evidence they find addresses the questions they are asking.

Those studies best suited to answering questions about benefits are randomised controlled trials, interrupted time series, and controlled before/after studies. All of these are characterised by the care taken to minimise the possibility that the measured effect of a policy is attributable to another factor which has not been measured (see Table 2 for an overview of the option information required and the associated study types). Very often policymakers will be able to find systematic reviews of these types of studies and this will save them a significant amount of time. In Article 7 in this series, we discuss how to find systematic reviews [9]. Once such systematic reviews have been found, policymakers then need to assess their quality and examine the findings in terms of their local applicability and their incorporation of considerations related to equity (see Table 3).

**Table 2:** Types of study designs well suited to providing particular types of information about options

Type of information about the option	Study designs well suited to providing the information	Definition
Benefits (i.e. positive effects)	Randomised controlled trials	• Experimental study in which individuals are randomly allocated to be exposed to different policy and programme options (e.g. using the toss of a coin or a list of random numbers generated by a computer)
	
	Interrupted time series	• Study using observations at multiple time points before and after a policy or programme is introduced (this is referred to as an 'interruption'). The design attempts to detect whether a policy or programme has had an effect significantly greater than any underlying trend over time
	
	Controlled before/after studies*	• Study in which observations are made before and after the implementation of a policy or programme, both in a group that is exposed to the policy or programme and in a control group that is not. Data collection is done concurrently in the two groups

Potential harms (i.e. negative effects)	Effectiveness studies (see above)	
	
	Observational studies	• Study in which observations are made about those exposed to a policy or programme. Data could be drawn from administrative databases, community surveys or other sources

Costs and cost-effectiveness	Cost-effectiveness studies	• Study in which the relative expenditures (costs) and outcomes (effects) of two or more courses of action are compared

Key elements of the option (how and why it works)	Qualitative studies carried out alongside a study of effects (i.e. process evaluations)	• Study conducted in natural settings and usually aimed at interpreting or making sense of phenomena in terms of the meanings people bring to them. Typically, narrative data are collected from individuals or groups of 'informants' (through interviews, focus groups, participant observation) or from documents. These are then interpreted by researchers

Views and experiences of stakeholders	Qualitative studies	• See above
	
	Observational studies	• See above

* These studies can be very time-consuming to find yet provide little information of value. This is due to the strong likelihood that those who have been exposed to an option, and those who have not been exposed to the option, differ in important ways. Impacts may be attributable therefore to differences between the groups rather than to differences in exposure to a particular option

**Table 3:** Issues to consider when assessing research evidence about the benefits, harms, and costs of options

Issue	Why it is important to consider the issue	Source of additional information
Quality	• Research evidence of low quality (i.e. that is not valid, credible or rigorous) can give policymakers a false impression of the likely costs and consequences of an option	• Article 8 in this series addresses how to assess the quality of systematic reviews [16] Article 16 addresses how to use a balance sheet incorporating assessments of the quality of evidence [17]

Applicability	• Research evidence produced in other jurisdictions can be valuable, but policymakers need to consider how likely it is that the costs and consequences of an option would be different in their setting	• Article 9 in this series addresses how to assess the applicability of the findings from systematic reviews to a specific setting [18]

Equity	• Research evidence focused on overall effects or effects *among *advantaged groups can be valuable. However, policymakers need to consider how likely it is that the costs and consequences of an option would be different in disadvantaged groups	• Article 10 addresses how to take equity into consideration when assessing the findings of a systematic review [19]

### 3. What harms are important to those who will be affected and which harms are likely with each option?

In this next step, the likely harms (or negative effects) of each option are determined. Again, policymakers will need to decide which harms are likely to be important to those who will be affected by the decisions they take. Some studies may address very infrequent outcomes such as death. Others may address frequent outcomes like the minor side effects of a drug, or focus on 'intermediate' outcomes such as the abandonment of routine tasks by lay health workers who have been asked to take on a new task. The 'POCO' acronym referred to earlier may also be used to structure searches for evidence of harms.

The types of studies best suited to answering questions about harms are more diverse. Information about harms can sometimes be derived from effectiveness studies. But more frequently, information can be found in observational studies that track those 'exposed' to an option, whether or not the exposure was part of a particular test of the option (e.g. a large-scale drug surveillance system). The pros and cons of these different data sources have been outlined elsewhere [10]. Policymakers will sometimes be able to find systematic reviews of these types of studies and will need to assess their quality and applicability, as well as incorporate equity considerations. (See Article 7 for further information on finding systematic reviews [9].) Local evidence about harms may also be found by policymakers and this issue is discussed in Article 11 in this series [11]. Once potential harms have been identified, the next step is then to identify what, if any, mitigating actions can be taken to reduce these harms.

### 4. What are the local costs of each option and is there local evidence about their cost-effectiveness?

The next step in characterising the costs and consequences of the options is to determine each option's costs and, if possible, its relative cost-effectiveness. Two options may both be effective but one may deliver better outcomes for a given cost, or it may achieve the same outcomes at less cost. Article 12 in this series addresses ways in which research evidence about resource use and costs can be incorporated in the assessment of options [12]. In Article 12 we discuss how data about costs need to be collected in the setting where the options are being considered. And it includes a discussion on how research evidence about cost-effectiveness is often limited by a lack of rigour in estimating effects, as well as by challenges in interpreting the valuation of resources being used, and by the 'black box' nature of the modelling.

Economic evaluations can often provide a useful framework for thinking through issues related to cost-effectiveness - even if policymakers need to treat the results of any given economic evaluation cautiously, just as they would for other types of studies. Economic evaluations, it should be remembered, are always written from a particular perspective, whether it be that of a provider, a payer, or of society at large. Policymakers and other stakeholders need to be aware of the particular viewpoint they themselves adopt for any given economic analysis.

### 5. What adaptations might be made to any given option and could they alter its benefits, harms and costs?

The penultimate step in characterising the costs and consequences of an option is to determine whether there might be significant interest in, or pressure, to *adapt *an option that has been tried elsewhere. In this instance, policymakers need to search specifically for qualitative studies (sometimes called *process evaluations*) carried out alongside studies of effects. Such studies can help to identify how and why an option works. These assessments can then be used to inform judgements as to whether particular elements of an option are critically important (and hence need to be retained), and which elements of an option are not important (and hence could be either dropped or modified). Article 4 in this series provides tips for finding qualitative studies [2].

### 6. Which stakeholder views and experiences might influence an option's acceptability and its benefits, harms and costs?

The final step in characterising the costs and consequences of options is to determine whether the views and experiences of stakeholders could influence the acceptability and impact of the options. Stakeholders may include healthcare recipients and citizens, healthcare providers, managers working in healthcare organisations, and policymakers. If influence is likely, then policymakers and those who support them need to seek out qualitative studies that specifically examine the views and experiences of such stakeholders. (Again, Article 4 in this series outlines tips related to finding qualitative studies) [2].

Table 4 provides guidance on identifying different types of research evidence.

**Table 4:** Finding research evidence about options

Characterising the costs and consequence of options involves finding and using many types of research evidence. When available, systematic reviews (the subject of Article 7) can help to characterise the benefits, harms, and key features of the options, as well as the views and experiences of stakeholders [9]. In the absence of systematic reviews, single studies must be found. Economic evaluations can help to characterise the cost-effectiveness of the options. The first set of steps involved in finding such reviews and studies includes:	
• Drawing up a list of words or phrases that capture the option (e.g. replacing 'health professionals who currently prescribe an anti-malarial drug' with 'lay health workers'), synonyms for each option (e.g. substitution), and alternative spellings for each option (e.g. doctor, doctors, physician, physicians, medical, medicine)	
• Deciding whether systematic reviews or single research studies are the focus of the search, and	
• Providing any additional details that limit the search (e.g. children, adults) The second set of steps includes:	
• Choosing those words and phrases that would *all *need to be present in order for the article to be identified (e.g. substitution, lay heath worker, and systematic review), connecting them with 'and', and placing each term in brackets	
• Choosing those words and phrases for which *only one *would need to be present (e.g. physician and its synonyms), connecting them with 'or', and putting each term in brackets, and	
• Connecting sets of brackets using 'and'	
The third set of steps includes:	
• Opening in an Internet browser the relevant database:	
◦ Program in Policy Decision-making/Canadian Cochrane Network and Centre (PPD/CCNC) database http://www.researchtopolicy.ca/search/reviews.aspx for systematic reviews of studies about health system arrangements (benefits, harms, key features, and stakeholders' views and experiences) - see Article 7 for additional information [9]	
◦ Cochrane Library's Cochrane Database of Systematic Reviews (CDSR) and Database of Abstracts of Reviews of Effects (DARE) http://thecochranelibrary.com for systematic reviews of programmes, services and drugs (benefits and possibly harms) - see Article 7 for additional information [9]	
◦ Cochrane Library's Economic Evaluation Database (EED) http://thecochranelibrary.com for economic evaluations	
◦ PubMed http://www.ncbi.nlm.nih.gov/pubmed for the 'hedges' (i.e. validated search strategies) to find specific types of single studies (harms, key features, and views and experiences of stakeholders) - see Article 4 for additional information [2]	
• Entering the words and phrases, as well as the Boolean operators 'and'/'or' in the search field, and	
• Clicking the appropriate icon to initiate the search	

## Conclusion

An appropriate set of options for a specific local context can be identified by combining creative thinking with generic taxonomies (like the one used to organise the PPD/CCNC database) or frameworks specific to a given issue or domain, or else bundling these combinations together. Each option can be assessed in terms of its likely local benefits, harms and costs or cost-effectiveness, and in terms of whether any adaptations might alter these benefits, harms and costs and related stakeholder views and experiences. Policymakers should take into account their quality and the local applicability of their findings when using systematic reviews to answer questions about benefits, harms and costs. They should also consider key equity considerations. These considerations form the focus of the following articles in this series: Article 8 (on assessing the quality of a systematic review), Article 9 (on assessing the local applicability of the findings of a systematic review) and Article 10 (on taking equity into consideration when assessing the findings of a systematic review). Policymakers should also be aware that they will face a practical challenge in assessing the relative value of the benefits, harms and costs and in making trade-offs between them. This topic is the focus of Article 16 which discusses the use of research evidence in balancing the pros and cons of different options.

## Resources

### Useful documents and further reading

- Kingdon JW: *Agendas, Alternatives, and Public Policies*, 2 edn. New York, USA: Longman; 2003, pp. 116-144

### Links to websites

- Program in Policy Decision-making (PPD)/Canadian Cochrane Network and Centre (PPD/CCNC) database:http://www.researchtopolicy.ca/search/reviews.aspx - Source of systematic reviews of studies about health system arrangements (benefits, harms, key features, and the views and experiences of stakeholders).

- Cochrane Library's Cochrane Database of Systematic Reviews (CDSR) and Database of Abstracts of Reviews of Effects (DARE): http://thecochranelibrary.com - Source of systematic reviews of programmes, services and drugs (benefits and possibly harms)

- Health-evidence.ca: http://health-evidence.ca - Source of systematic reviews of public health programmes and services (benefits and possibly harms).

- Cochrane Library's Economic Evaluation Database (EED): http://thecochranelibrary.com - Source of economic evaluations.

- PubMed:http://www.ncbi.nlm.nih.gov/pubmed - Source of 'hedges' (i.e. validated search strategies) to find select types of single studies (harms, key features, and the views and experiences stakeholders).

- BIREME's Virtual Health Library:http://www.virtualhealthlibrary.org/php/index.php?lang=en - Source for many research products and databases available in the languages spoken in the Americas (Spanish and Portuguese primarily as well as English and French).

## Competing interests

The authors declare that they have no competing interests.

## Authors' contributions

JNL prepared the first draft of this article. MGW, ADO, JG, SL and AF contributed to drafting and revising it.

## Supplementary Material

Additional file 1 Glossary Click here for file
